# Supplementary material for: Critical Role of Neuronal Vps35 in Blood Vessel Branching and Maturation in Developing Mouse Brain
Source: Biomedicines. 2022 Jul 9;10(7):1653. doi: 10.3390/biomedicines10071653 (PMC9313219; doi:10.3390/biomedicines10071653)
Supplement: Supplementary file 1 [file biomedicines-10-01653-s001.zip › Supplementary figure legends.docx]

**Figure S1.** Reduced PECAM-1^+^ vessels in *Vps35^GFAP^* cortex and hippocampus. **(A)** *Vps35^f/f^* mice were crossed with GFAP Cre mice to generate *Vps35^GFAP^* mutant mice. **(B)** Western blot analysis of Vps35 level in cortex and hippocampus taken from *Vps35^f/f^* and *Vps35^GFAP^* animals. β-actin was employed as a loading control. **(C)** Quantification analysis of relative Vps35 protein expression level from B (n=3 animals per genotype; two-tailed unpaired t test). **(D)** Representative images of BVs in the S1 cortex of control and *Vps35^GFAP^* mice at age of P14. Brain sections were subjected to immunostaining analysis using antibodies PECAM-1 (red). **(E)** Representative images of BVs in the hippocampus and striatum of control and *Vps35^GFAP^* mice at age of P14. Brain sections were subjected to immunostaining analysis using antibodies PECAM-1 (red). **(F)** Higher-magnification images of the boxed regions of D. **(G)** Quantification of PECAM-1^+^ vessel length and BV branches in E, F (n=3 mice per genotype; two-tailed unpaired t test). Scale bars as indicated in each panel. Individual data points were shown as dots with group mean ± S.E.M; **p<0.01; ****p<0.0001; ns., not significant.

**Figure S2.** Reduced PECAM-1^+^ vessels in *Vps35^Emx1^* cortex and hippocampus. **(A)** *Vps35^f/f^* mice were crossed with Emx1 Cre mice to generate *Vps35^Emx1^* mutant mice. **(B)** Western blot analysis of Vps35 level in cortex and hippocampus taken from *Vps35^f/f^* and *Vps35^Emx1^* animals. β-actin was employed as a loading control. **(C)** Quantification analysis of relative Vps35 protein expression level from B (n=3 animals per genotype; two-tailed unpaired t test). **(D)** Representative images of blood vessels (BVs) in the cortex of control and *Vps35^Emx1^* mice at age of P14. Brain sections were subjected to immunostaining analysis using antibodies PECAM-1 (red). **(E)** Higher-magnification images of the boxed regions of D and BV tracing of boxed regions. **(F)** Quantification of PECAM-1^+^ vessel length and BV branches in E (n=3 mice per genotype; two-tailed unpaired t test). **(G)** Representative images of BVs in the hippocampus of control and *Vps35^Emx1^* mice at age of P14. Brain sections were subjected to immunostaining analysis using antibodies PECAM-1 (red), the nuclei were stained with DAPI (blue). **(H)** Higher-magnification images of the boxed regions of G and BV tracing of boxed regions. **(I)** Quantification of PECAM-1^+^ vessel length and BV branches in H (n=3 mice per genotype; two-tailed unpaired t test). Scale bars as indicated in each panel. Individual data points were shown as dots with group mean ± S.E.M; **p<0.01; ***p<0.001; ****p<0.0001.

**Figure S3.** Comparable PECAM-1^+^ vessels in *Vps35^Camk2a^* cortex and hippocampus. **(A)** Representative images of blood vessels (BVs) in the cortex of control and *Vps35^Camk2a^* mice at age of 2M. Brain sections were subjected to immunostaining analysis using antibodies PECAM-1 (red). Higher-magnification images of the boxed regions were shown in lower panels. **(B)** Representative images of BVs in the hippocampus of control and *Vps35^Camk2a^* mice at age of 2M. Brain sections were subjected to immunostaining analysis using antibodies PECAM-1 (red), the nuclei were stained with DAPI (blue). Higher-magnification images of the boxed regions were shown in offside panels. **(C, D)** Quantification of PECAM-1^+^ vessel length in A and B (n=3 mice per genotype; two-tailed unpaired t test). Scale bars as indicated in each panel. Individual data points were shown as dots with group mean ± S.E.M; ns., not significant.

**Figure S4.** Altered GFAP^+^ astrocyte distribution in *Vps35^Neurod6^* hippocampus. **(A)** Representative images of hippocampal brain sections co-immunostained with PECAM-1, SMA and GFAP at P14. **(B)** Higher-magnification images of the boxed regions in A. **(C)** Quantification analysis of PECAM-1^+^/SMA^+^ BV associated with GFAP (n=3 mice per group; two-tailed unpaired t test). **(D)** Representative images of hippocampal brain sections co-immunostained with PECAM-1, SMA and GFAP at P21. **(E)** Higher-magnification images of the boxed regions in D. **(F)** Quantification analysis of PECAM-1^+^/SMA^+^ BV associated with GFAP (n=3 mice per group; two-tailed unpaired t test). **(G-H)** Representative images of hippocampal brain sections co-immunostained with PECAM-1 and GFAP at P14/P21, the nuclei were stained with DAPI (blue). **(I)** Quantification analysis of the distribution of GFAP+ cells between DG and CA1 GFAP (n=3 mice per group). Scale bars as indicated in each panel. Individual data points were shown as dots with group mean ± S.E.M; *p<0.05; **p<0.01; ns., not significant.

**Figure S5.** Comparable arteriole diameter and GFAP^+^ astrocyte distribution in *Vps35^Neurod6^* cortex at P7. **(A)** Representative images of cortical brain sections co-immunostained with PECAM-1, SMA and GFAP at P7. **(B)** Quantification analysis of arteriole diameter and relative GFAP intensity of control and *Vps35^Neurod6^* mice (n=3~4 mice per group; two-tailed unpaired t test). Scale bars as indicated in each panel. Individual data points were shown as dots with group mean ± S.E.M; ns., not significant.

**Figure S6.** Quantification of mRNA expression in the P14 brain. **(A-D)** Analysis of *Vps35* and gene of classic angiogenic factors mRNA levels in cortex and hippocampus from control and *Vps35^Neurod6^* mice (n=3~4 mice per group; two-tailed unpaired t test). Individual data points were shown as dots with group mean ± S.E.M; *p<0.05; ns., not significant.
