# Supplementary figures and images for: Critical Role of Neuronal Vps35 in Blood Vessel Branching and Maturation in Developing Mouse Brain
Source: Biomedicines. 2022 Jul 9;10(7):1653. doi: 10.3390/biomedicines10071653 (PMC9313219; doi:10.3390/biomedicines10071653)

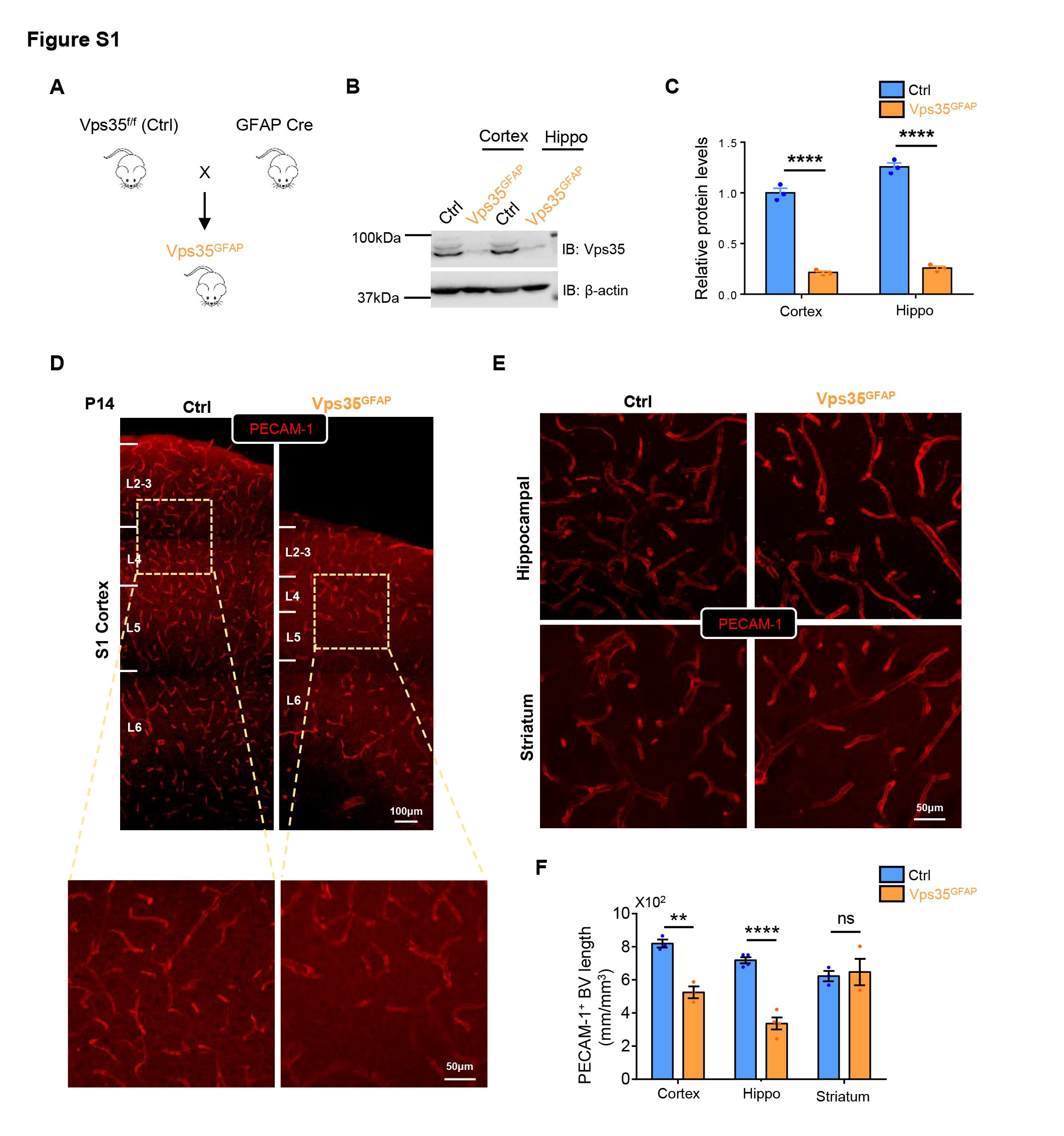

Supplement: Supplementary file 1 [file biomedicines-10-01653-s001.zip › Figure-S1.tif]

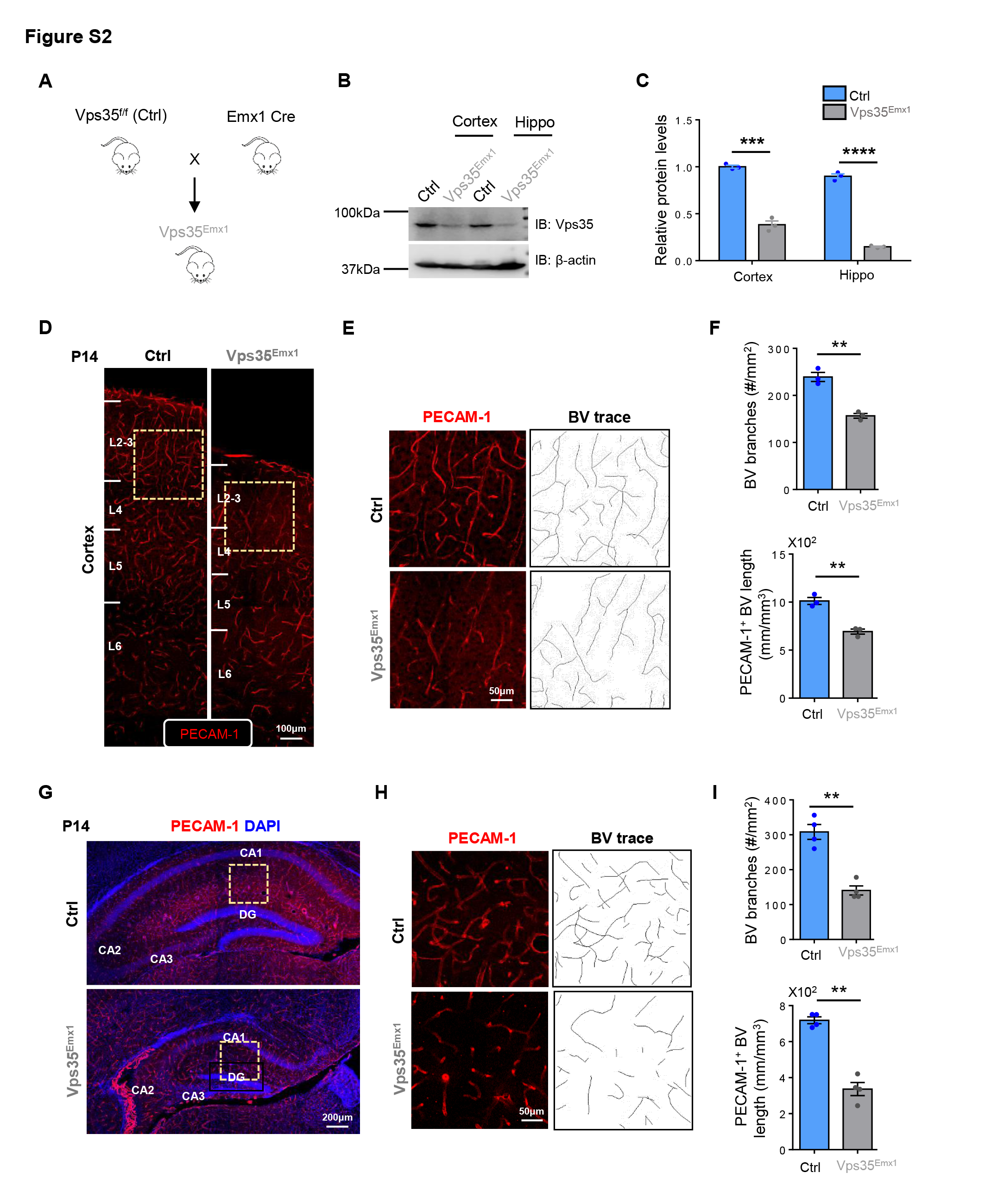

Supplement: Supplementary file 1 [file biomedicines-10-01653-s001.zip › Figure-S2.tif]

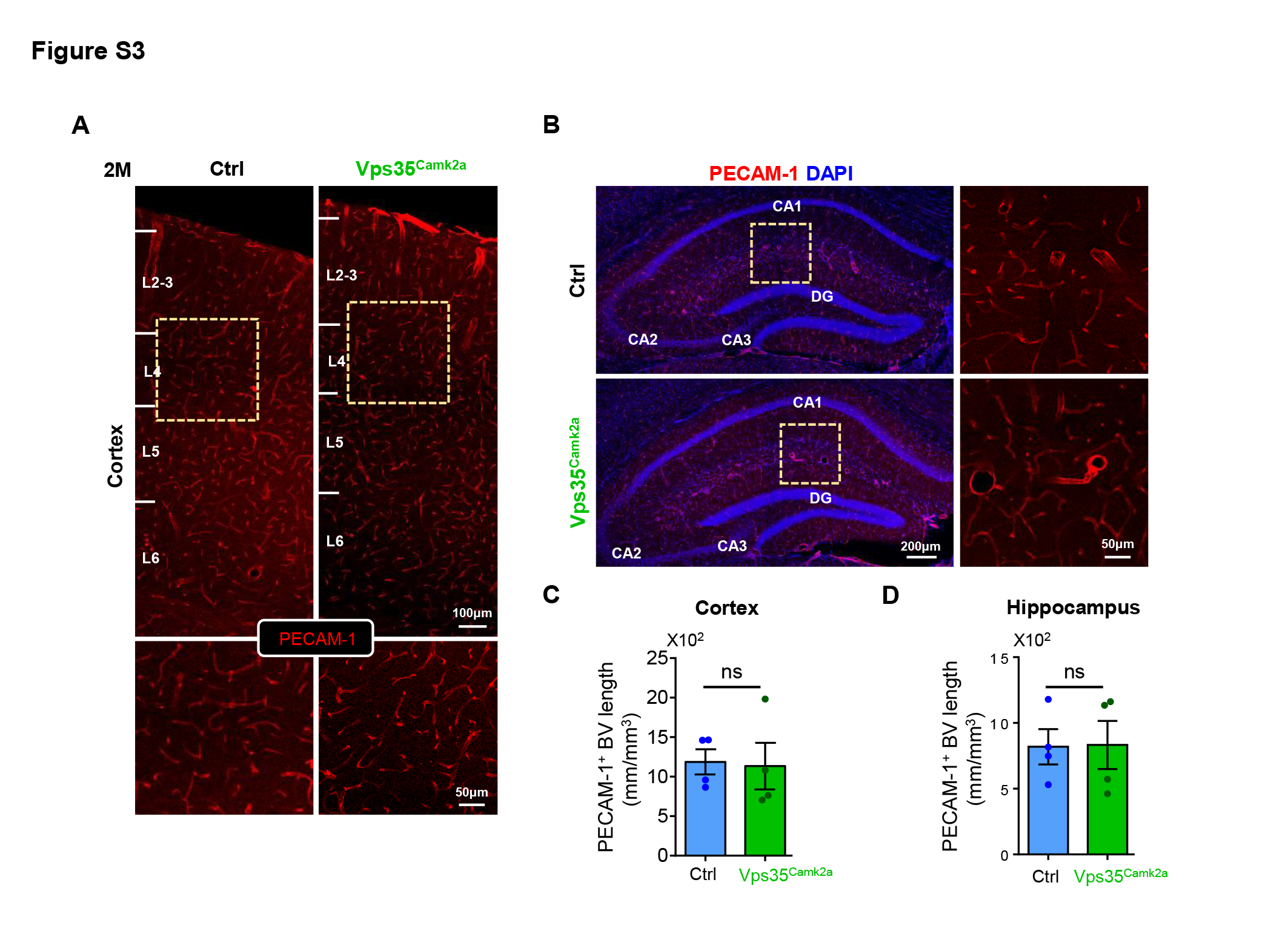

Supplement: Supplementary file 1 [file biomedicines-10-01653-s001.zip › Figure-S3.tif]

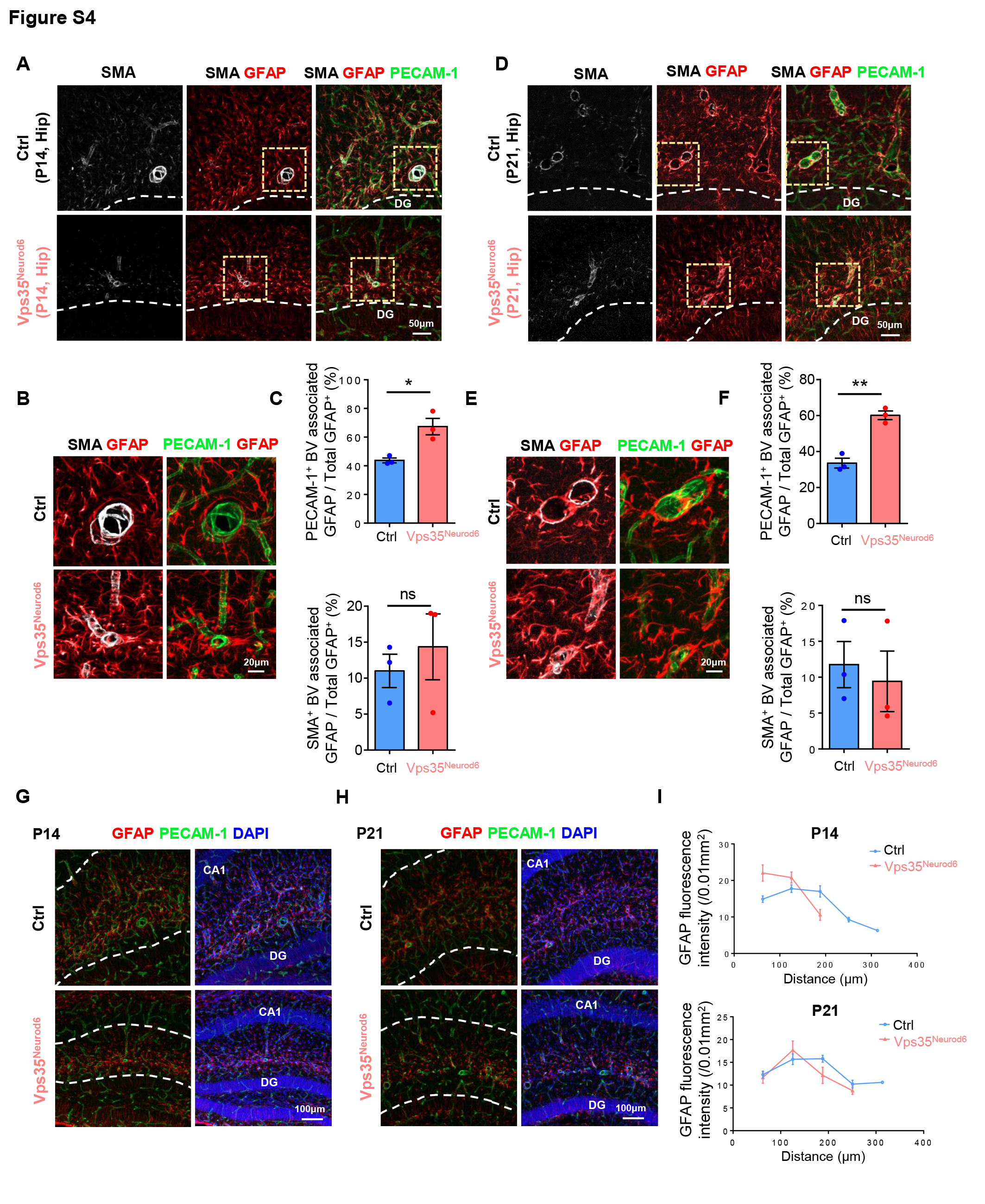

Supplement: Supplementary file 1 [file biomedicines-10-01653-s001.zip › Figure-S4.tif]

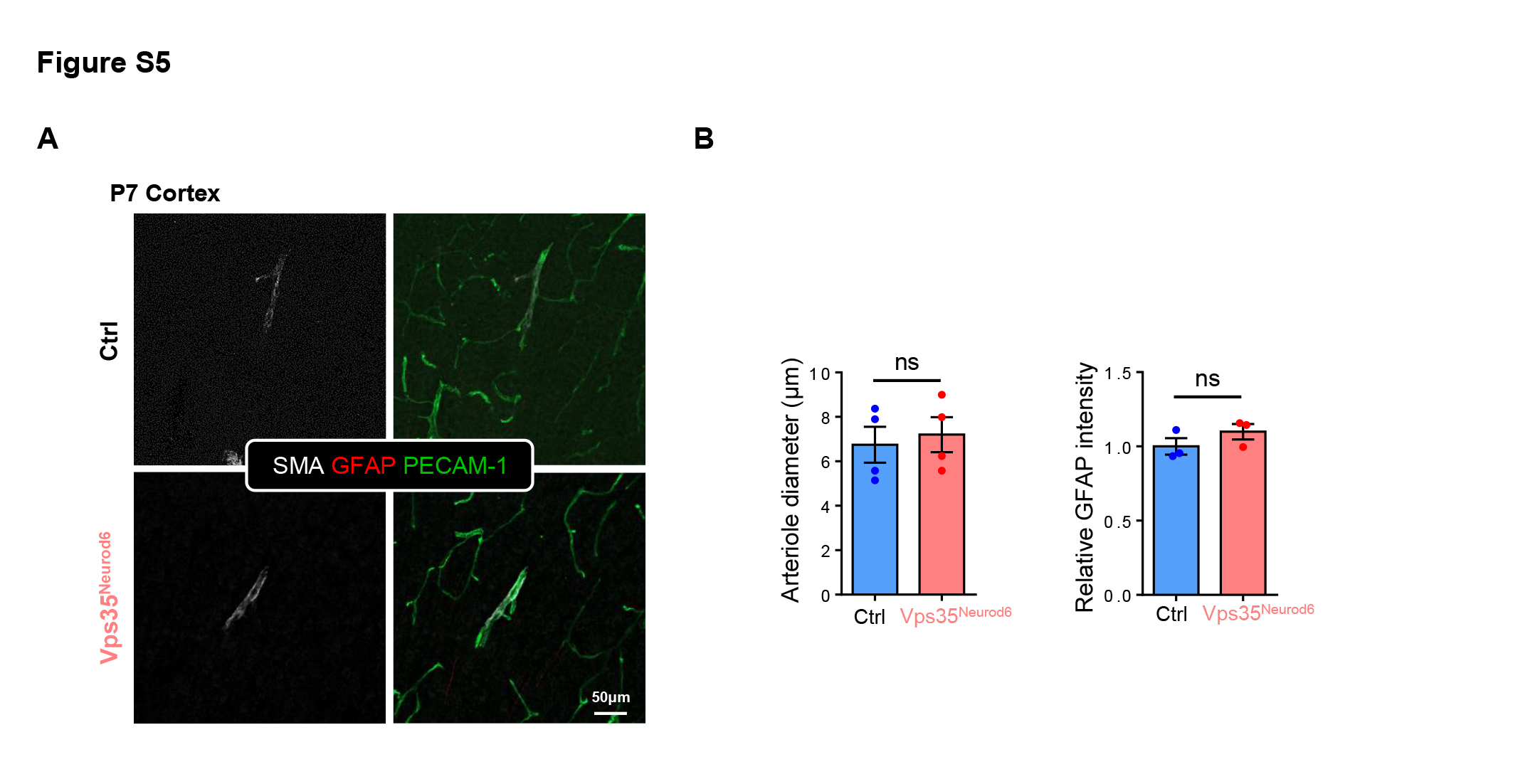

Supplement: Supplementary file 1 [file biomedicines-10-01653-s001.zip › Figure-S5.tif]

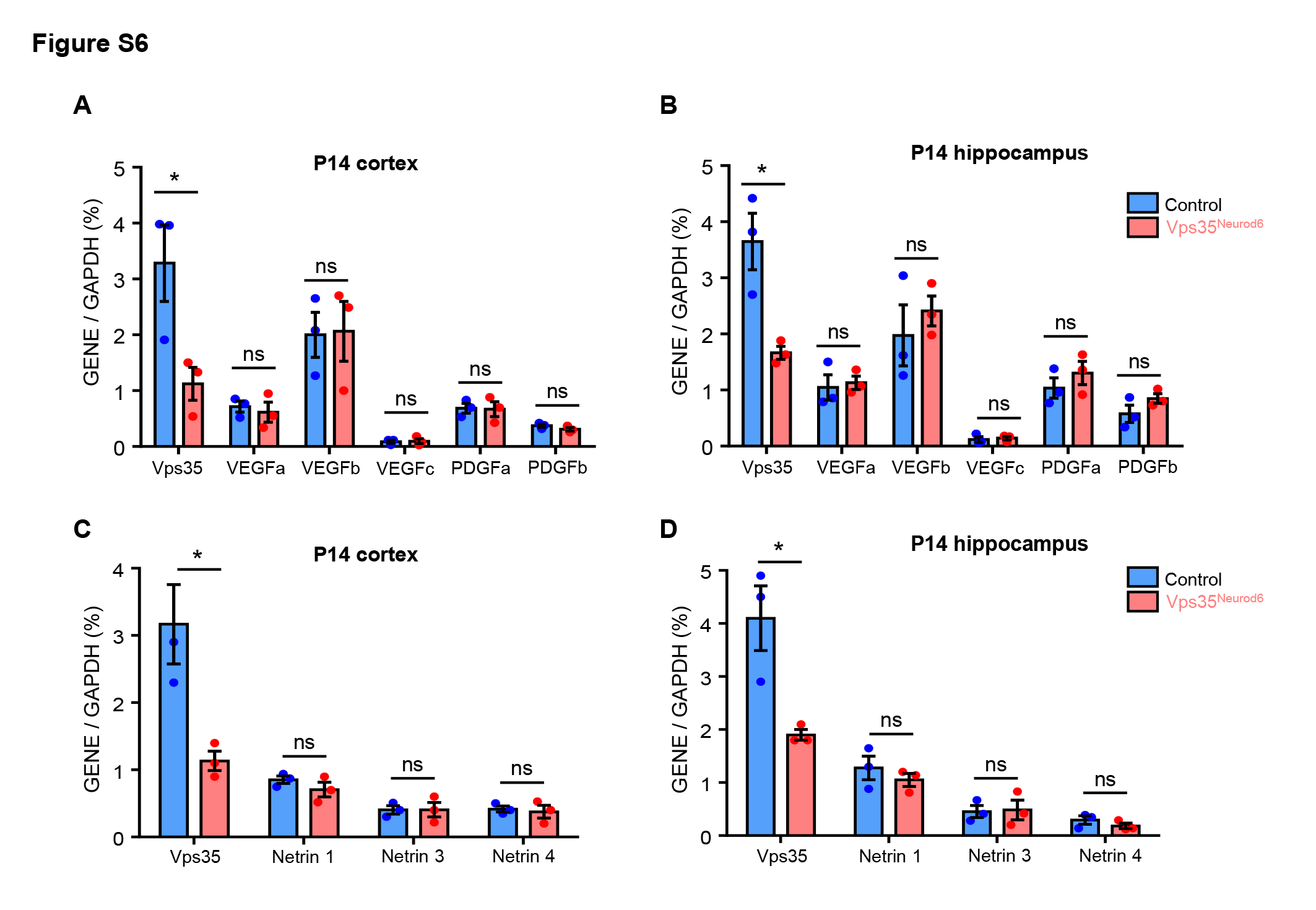

Supplement: Supplementary file 1 [file biomedicines-10-01653-s001.zip › Figure-S6.tif]
